# A stochastic simulation model to study respondent-driven recruitment

**DOI:** 10.1371/journal.pone.0207507

**Published:** 2018-11-15

**Authors:** Mart L. Stein, Vincent Buskens, Peter G. M. van der Heijden, Jim E. van Steenbergen, Albert Wong, Martin C. J. Bootsma, Mirjam E. E. Kretzschmar

**Affiliations:** 1 National Coordination Centre for Communicable Disease Control, Centre for Infectious Disease Control, National Institute for Public Health and the Environment, Bilthoven, Utrecht, The Netherlands; 2 Department of Sociology, Faculty of Social and Behavioural Sciences, University Utrecht, Utrecht, The Netherlands; 3 Department of Methodology and Statistics, Faculty of Social and Behavioural Sciences, University Utrecht, Utrecht, The Netherlands; 4 Southampton Statistical Sciences Research Institute, University of Southampton, Southampton, United Kingdom; 5 Centre of Infectious Diseases, Leiden University Medical Centre, Leiden, The Netherlands; 6 Department of Statistics, Informatics and Mathematical Modelling, National Institute for Public Health and the Environment, Bilthoven, The Netherlands; 7 Julius Center for Health Sciences and Primary Care, University Medical Center Utrecht, Utrecht, The Netherlands; 8 Department of Mathematics, Faculty of Sciences, Utrecht University, Utrecht, The Netherlands; 9 Centre for Infectious, Disease Control, RIVM, Bilthoven, Utrecht, The Netherlands; The University of Hong Kong, CHINA

## Abstract

Respondent-driven detection is a chain recruitment method used to sample contact persons of infected persons in order to enhance case finding. It starts with initial individuals, so-called seeds, who are invited for participation. Afterwards, seeds receive a fixed number of coupons to invite individuals with whom they had contact during a specific time period. Recruitees are then asked to do the same, resulting in successive waves of contact persons who are connected in one recruitment tree. However, often the majority of participants fail to invite others, or invitees do not accept an invitation, and recruitment stops after several waves. A mathematical model can help to analyse how various factors influence peer recruitment and to understand under which circumstances sustainable recruitment is possible. We implemented a stochastic simulation model, where parameters were suggested by empirical data from an online survey, to determine the thresholds for obtaining large recruitment trees and the number of waves needed to reach a steady state in the sample composition for individual characteristics. We also examined the relationship between mean and variance of the number of invitations sent out by participants and the probability of obtaining a large recruitment tree. Our main finding is that a situation where participants send out any number of coupons between one and the maximum number is more effective in reaching large recruitment trees, compared to a situation where the majority of participants does not send out any invitations and a smaller group sends out the maximum number of invitations. The presented model is a helpful tool that can assist public health professionals in preparing research and contact tracing using online respondent-driven detection. In particular, it can provide information on the required minimum number of successfully sent invitations to reach large recruitment trees, a certain sample composition or certain number of waves.

## Introduction

Many infectious diseases are transmitted via close or intimate contact between individuals. By sampling contact persons of infected persons in a network, one can study transmission routes of these pathogens within networks and to detect hitherto hidden cases. Such information is important for effective control of disease outbreaks. Respondent-driven detection (RDD), a method derived from snowball sampling, is a chain recruitment method that allows for sampling of contact persons of participants [1, 2]. With RDD, initial individuals (or ‘seeds’) are invited for participation, which includes filling in a questionnaire. At the end of the questionnaire, seeds receive a fixed number of invitation coupons (usually four, according to standardised methodology of respondent-driven sampling (RDS) [3]), and are asked to invite a number of contact persons whom they have met during a specific time period. Recruitees (i.e., invitees who participate) are then asked to do the same, resulting in successive ‘waves’ of contact persons. Unlike with snowball sampling, each coupon contains a personal code with which peer recruitment is followed. A set of participants connected via recruitment links to one seed is referred to as a ‘recruitment tree’.

The composition of the sample consisting of all participants who completed the survey depends on how likely individuals with certain characteristics are recruited. For instance, individuals with a high probability of being recruited will likely be overrepresented in the sample. For RDS, statistical techniques are available to estimate population characteristics from the respondent-driven sample [4–6]. Most of these techniques are based on the assumption of a first-order Markov chain process, i.e., correlations found between recruiters and recruitees are only dependent on the direct recruiter [7]. Given that seeds may be chosen non-randomly, and that people tend to have contact with individuals with similar characteristics [8], the characteristics of the participants in the first few recruitment waves may still reflect the characteristics of the seeds [9]. If peer recruitment proceeds through a sufficiently large number of waves, recruitees with different characteristics enter the sample. With increasing number of waves, the composition of the sample will converge to a stable distribution and become independent of the (often) non-randomly selected seeds [3, 10, 11]. Although the primary objective of RDD is to detect cases, rather than to estimate population proportions from samples, we were interested in how fast equilibrium is reached under different mixing conditions, e.g., random recruitment versus recruitment with a preference.

Previously, we conducted surveys to investigate the feasibility of online RDD to study contact patterns that are relevant for the transmission of respiratory pathogens and to exploit the use of the network of cases to detect other cases [1, 2]. However, in those studies, the numbers of recruitees via online peer recruitment were low. The majority of participants did not or not fully comply with sending invitations, and a limited number of invitees actually participated. Therefore, peer recruitment stopped after a few waves. This problem is common with respondent-driven studies [12]. To improve case finding with online RDD, or obtain a required number of recruitees or a required sample composition, it is key to find ways to increase rates of peer recruitment, and to understand under which circumstances sustainable recruitment is possible.

In the literature, there are many examples of models used to study recruitment behaviour with RDS or snowball sampling (see e.g., [13–18]). Malmros et al. (2014) used a configuration model, without empirical data, to analyse the influence of the number of coupons and probability of coupon transfer between a recruiter and recruitee on the recruitment process [13]. To our knowledge, there is one simulator of the recruitment process available in the public domain; this model allows for only a limited number of factors influencing recruitment, and only reports aggregated numbers of recruitees [19]. Mathematically, the recruitment process can be described by a multitype branching process, and results from the theory of branching processes can be applied (e.g. [20]).

Here, we used simulations of a multi-type branching process that included more factors to study the dynamics of a respondent-driven recruitment process. Simulating the recruitment process enabled us to analyse the influence of various factors on the final sample size and sample composition. We used empirical data to suggest parameter choices in the simulation model. Data were taken from a sample of recruitment trees collected with online RDD in the Netherlands during the winter season of 2013–2014 [1, 2]. We aimed to determine which factors are important for the success of recruitment, to identify thresholds for reaching sustainable recruitment in recruitment trees, and to determine how fast a steady state is reached in the sample composition for individual characteristics. Successful recruitment can be defined at two levels. At the level of a recruiter, successful recruitment means that an invitee also completes the questionnaire and becomes a recruitee. At the level of a recruitment tree, successful recruitment indicates that a tree continues to grow due to continued peer recruitment. The results of our analysis can be used to inform future online RDD surveys, e.g., for determining the mean number of successfully sent invitations per participant required to obtain on average growing recruitment trees.

## Methods

### A stochastic simulation model

We considered respondent-driven recruitment in a heterogeneous population. Individuals (i.e., recruiters and recruitees) were characterized by three categorical variables, namely sex, age groups, and education level. Starting from a seed, the recruitment process was modelled as a multi-type discrete time branching process. Number and type of recruitees of a recruiter in wave *W* depended on that recruiter’s characteristics, on the number of invitations he sent out, and the number of invitees who accepted the invitation, for all *W*. Each recruiter could send a maximum number of invitations *c*.

We assumed that the number of invitations per recruiter had a beta-binomial distribution, with shape parameters *α* and *ß* depending on the characteristics of the recruiter. The reason to choose a beta-binomial distribution was that it can reproduce bimodal distributions of numbers of invitations as observed in the data, with a peak at zero and another peak at the maximum value *c*. The latter reflects a restricted number of invitations per recruiter, which is common in RDS surveys. We then assumed that given a number of invitations, the number of accepted invitations had a binomial distribution where the probability of acceptance *p* depended on the characteristics of the recruiter. The characteristics of recruitees were dependent on the characteristics of the recruiter to reflect correlations between recruiter–recruitee pairs in their characteristics, but assuming independence of the three characteristics. We used the mean number of invitations sent out ($\bar{x}$) and the proportion accepted invitations (*p*) from our sample to calculate the mean number of successfully sent out invitations ($\bar{x}$ * *p*).

### Simulation model

Each model run started with one seed in wave 0. For each wave *W*, the model generated a list of individuals and their characteristics. Then, for each recruiter, the number of invitations sent out and the number of invitations accepted by invitees was determined based on random draws from probability distributions. Characteristics of new recruitees were determined based on correlations with characteristics of their recruiters. New recruitees then formed wave *W+1*. Each recruitee in the model received a unique identifier (a numerical string) that linked it to its recruiter. A model run stopped when no invitations were sent out, when no invitations were accepted, or when after a wave a total number N of recruitees was exceeded. In our simulations, the number N of recruitees, at which further recruitment was stopped, was set to N = 1000. If a recruitment tree became larger than 1000 recruitees, the tree showed ongoing recruitment and continued to grow if the model run was not stopped manually. The final recruitment tree consisted of all recruitees recruited during one simulation run. The model was implemented in R (R Foundation for Statistical Computing, Vienna, Austria) version 3.2.2 and is available online: https://github.com/SteinML/RDDmodel.git. For model formulation and details, we refer the reader to S1 Text.

### Outcome measures

To quantify the success of recruitment, we kept track of the number of recruitees in each recruitment tree starting with one seed (i.e., the size of recruitment trees). If the size of a recruitment tree was larger or equal than N = 1000, we labelled it as ‘large’. We then computed the proportion of runs with large recruitment trees for each set of parameters. The probability of obtaining large recruitment trees depends on the specific parameter combination. If participants successfully invite on average less than 1 new recruitee, only small recruitment trees occur, while above this threshold small and large recruitment trees occur. The proportion of large recruitment trees increases with increasing mean number of successfully sent invitations. We recorded for which parameter combinations at least 5% of runs resulted in a large recruitment trees. We also recorded the maximum wave number reached by each tree to quantify the length of recruitment trees.

To investigate the sample composition at equilibrium, we calculated for each simulation run the composition of each wave with respect to sex, age group and educational level. We used the standard criterion from the RDS literature to define equilibrium [3, 9]. This criterion states that sample proportions are viewed as stable when the relative difference between proportions in subsequent waves is less than two percent. To quantify the convergence of the composition in our simulations, we investigated at which wave equilibrium according to the above criterion was reached. Proportions were calculated *per wave* and averaged over simulation runs.

By way of illustration of how the model can provide guidance for future surveys, we also included influenza vaccine beliefs in the model. Beliefs about a vaccine can strongly affect individual vaccination decisions. In particular, negative vaccine beliefs can lead to low vaccination rates, which in turn can lead to higher likelihood of a disease outbreak [21].

### Model parameters

#### Study population

Model parameters were suggested by data collected during an online RDD study performed in the Netherlands during the winter season of 2013–2014 [1]. Participants were enrolled via a large web based participatory surveillance panel. After filling out a questionnaire, each participant received four unique electronic coupons to invite contact persons whom they had met in the previous two weeks. A total of 1015 volunteers entered the RDD survey as seeds, and 433 recruitees were successfully recruited. Recruitment reached up to 6 waves of recruitees. For each participant, sociodemographic variables were recorded, including sex (females and males), age (three age groups), education level (two categories) and vaccine belief (two categories: ‘positive’ and ‘negative or undecided’). Participants were allowed to fill in the survey for one of their children. Overall, participants had a mean age of 53.7 years (standard deviation (SD): 14.5 years; range: 3–97 years), 64.8% was female, 57.3% had an academic education (i.e., participants obtained a bachelor degree or higher in higher education) and 53.5% had a negative vaccine belief (see Table 1).

**Table 1:** Participants’ characteristics and recruitment behaviour as observed during RDD survey in the Netherlands.

Variables	Seeds in wave 0**	Recruitees in waves 1 to 6**	Overall (seeds + recruitees)*
Number of female participants (%)	662 (65.2%)	277 (64.0%)	939 (64.8%)
Mean age in years of participants (SD; range)	55.5 (13.0; 4–97)	49.4 (16.8; 3–82)	53.7 (14.5; 3–97)
Number of participants in age group 0–39, a1 (%)	127 (12.5%)	121 (27.9%)	248 (17.1%)
Number of participants in age group 40–59, a2 (%)	465 (45.8%)	173 (40.0%)	638 (44.1%)
Number of participants in age group 60+, a3 (%)	423 (41.7%)	139 (32.1%)	562 (38.8%)
Number of participants with an academic education, B^¥^	596 (58.7%)	234 (54.0%)	830 (57.3%)
Number of participants with a negative vaccine belief	482 (47.5%)	293 (67.7%)	775 (53.5%)
Mean number of invitations sent out, $\bar{x}$ (s^2^)	1.33 (3.24)	1.47 (3.23)	1.36 (3.21)
Mean proportion invitation was accepted, *p* (range)	18.9% (0%– 26.9%)	20.1% (7.7%– 31.6%)	19.2% (10.0%– 27.2%)

*A stratification by recruiter characteristics can be found in S1 Table.

**A stratification by recruiter characteristics *and* by seeds and recruitees can be found in S2 Table.

^¥^Academic education defined as acquired a bachelor degree or higher in higher education.

#### Recruitment behaviour

Of the 1448 participants (i.e., seeds and recruitees) who completed the survey, 609 (42.1%) sent out invitations. The overall mean number of invitations sent out was 1.36 (s^2^: 3.21). The distribution of the number of invitations sent out was bimodal, with a peak at zero and another peak at four. We fitted a beta-binomial distribution to the observed frequency distribution of invitations sent out by participants, using maximum likelihood estimation in the R package “VGAM” (function “betabinomialff”). The mean number of invitations sent out varied for different subgroups of participants depending on sex, age and educational level between 0.64 (s^2^: 2.02) and 1.67 (s^2^: 3.48) (see S1 Table). Furthermore, a difference in mean numbers of sent invitations between seeds and all recruitees was observed (see S1 Fig). Seeds sent on average slightly less invitations compared to recruitees, 1.33 (s^2^: 3.24) and 1.47 (s^2^: 3.23) respectively (see Table 1 and S2 Table). Female seeds, with an academic education in the age group 60 years and older, were most active with sending invitations to others.

We based the probability of an invitation being accepted in the model on the number of recruitees divided by total number of invitations sent out by recruiters with specific characteristics in the data set. The acceptance probability therefore depended on the characteristics of the recruiter, and not on the characteristics of the recruitee. Overall, 19.2% (range: 10.0% - 27.2% for different recruiter characteristics) of all invitations sent were accepted by recruitees. The mean proportion of acceptance only slightly differed between seeds (18.9%) and recruitees (20.1%). S2 Table displays an overview of the fitted beta binomial distributions and proportions of invitations accepted stratified by recruiter characteristics, and by seeds and recruitees.

#### Mixing parameters

The characteristics of the recruitees were determined based on random draws from probability distributions for sex, age and educational level, where the probability distributions depended on characteristics of the recruiter and were assumed to be independent for the three characteristics. Overall, the data showed that participants tended to recruit recruitees with similar characteristics. This is reflected by correlation coefficients for recruiter-recruitee pairs by characteristic [22]. A positive correlation (>0.10) reflects assortative mixing (i.e., inviting recruitees with the same characteristics), while a negative correlation (< -0.10) reflects disassortative mixing (i.e., inviting recruitees with other or the opposite characteristics). A correlation between -0.10 and 0.10 is usually interpreted as random mixing, which indicates that participants do not have a tendency to recruit recruitees with specific characteristics. Participants recruited mainly recruitees of a similar age group (*r*_*rank*_: 0.33 (0.25–0.42)) and with a similar education (*r*_*φ*_: 0.31 (0.22–0.39)). We based the probability distributions for sex, age and educational level on the proportions observed in the data set, e.g., the proportion of female participants who invited female recruitees. As the mixing behaviour varied over waves (e.g., recruiters in wave 1 invited more females and recruitees with an academic education, as compared to seeds in wave 0), we calculated both overall mixing proportions and mixing proportions stratified by waves (see Table 2).

**Table 2:** Heterogeneity in recruitment mixing behaviour over waves as observed during RDD survey in the Netherlands.

Mixing	Proportions waves 0–1 (n: 295)	correlation	Proportions waves 1–2 (n: 86)	correlation	Proportions waves 2 to 6 (n: 52)	correlation	Mean overall proportions (n: 433)	Overall correlations (n: 433)
Female–Female	0.61	0.02 [-0.09–0.14]	0.73	0.16 [-0.05–0.36]	0.83	0.23 [-0.05–0.47]	0.66	0.08 [-0.01–0.17]
Female—Male	0.39		0.27		0.17		0.33	
Male–Male	0.41		0.43		0.50		0.42	
Male–Female	0.59		0.57		0.50		0.58	
Age 0–39 (a1)–Age 0–39 (a1)	0.56	0.23 [0.12–0.34]	0.57	0.52 [0.34–0.66]	0.86	0.65 [0.46–0.78]	0.60	0.33 [0.25–0.42]
Age 0–39 (a1)–Age 40–59 (a2)	0.12		0.29		0.14		0.18	
Age 0–39 (a1)–Age 60+ (a3)	0.31		0.14		0.00		0.22	
Age 40–59 (a2)–Age 0–39 (a1)	0.28		0.19		0.20		0.26	
Age 40–59 (a2)–Age 40–59 (a2)	0.51		0.67		0.67		0.56	
Age 40–59 (a2)–Age 60+ (a3)	0.21		0.14		0.13		0.19	
Age 60+ (a3)–Age 0–39 (a1)	0.23		0.10		0.07		0.19	
Age 60+ (a3)–Age 40–59 (a2)	0.29		0.21		0.20		0.26	
Age 60+ (a3)–Age 60+ (a3)	0.49		0.69		0.73		0.55	
Lower than academic education (A)—Lower than academic education (A)	0.65	0.29 [0.18–0.39]	0.67	0.41 [0.22–0.57]	0.68	0.21 [-0.06–0.46]	0.65	0.31 [0.22–0.39]
Lower than academic education (A)—Academic education (B)	0.35		0.33		0.31		0.35	
Academic education (B)—Academic education (B)	0.65		0.76		0.53		0.66	
Academic education (B)—Lower than academic education (A)	0.35		0.24		0.47		0.34	

We assumed that vaccine belief is determined by individual characteristics and does not influence the recruitment process. We used a logistic regression model to estimate the probabilities of having a positive or negative belief about the influenza vaccine depending on age, sex, and education. Vaccine beliefs of individuals were determined based on random draws from the estimated probability distributions (see S1 Text and S4, S5 and S6 Tables). The observed time between sending of invitations by a recruiter and the moment of acceptance by their recruitees was not related to the characteristics of the recruiter, and therefore not included in the scenario analyses.

#### Scenarios

In total, we defined and investigated 18 scenarios (see Table 3), which were distinct in their parameter choices. For each parameter combination, starting with one seed, we performed 100 simulation runs. In scenario S1, we explored the relation between mean (*μ*) and variance (*σ*^*2*^) of the beta-binomial distribution for the number of invitations, and the probability of obtaining large recruitment trees. We randomly drew 9000 combinations of *μ* and *σ*^*2*^, ran the simulations and recorded the proportion of large trees per parameter combination. We assumed a probability of acceptance of invitations of 1.

**Table 3:** Defined scenarios with distinct parameter choices used in simulation runs.

			Parameters													
			Beta-binomial distribution			Proportion accepts invitation	Mixing by sex, proportion		Mixing by age groups, proportion						Mixing by education level, proportion	
Scenarios		Seeds	*c*	*μ*	*σ*^*2*^	*p*	F-F	M-M	a1-a1	a1-a2	a2-a1	a2-a2	a3-a1	a3-a2	A-A	B-B
Randomly draw 9000 combinations of ***μ*** and ***σ***^***2***^	S1	1	4	[0.1–3.9]	[0.1–3.9]	1	Successful sending was not dependent on characteristics recruiter, thus no influence of mixing behaviour on number of recruitees in recruitment trees.									
***μ*** and ***p*** depend on recruiter’s characteristics	S2	1015 from data	4	See S1 Table	See S1 Table	See S1 Table	0.66	0.42	0.60	0.18	0.26	0.56	0.19	0.26	0.65	0.66
***μ*** and ***p*** depend on recruiter’s characteristics + whether seed or recruitee	S3	1015 from data	4	See S2 Table	See S2 Table	See S2 Table	0.66	0.42	0.66	0.18	0.26	0.56	0.19	0.26	0.65	0.66
***μ*** and ***p*** depend on recruiter’s characteristics + whether seed or recruitee, assuming heterogeneity mixing behaviour over waves	S4	1015 from data	4	See S2 Table	See S2 Table	See S2 Table	Assumed heterogeneity in mixing behaviour for different waves, as observed in the data. Values taken from Table 2.									
Assuming a beta-binomial distribution with highest possible ***σ***^***2***^ (as observed in data)	S5	1	4	0.1, 0.2,…, 3.9	0.1–3.4*	0.19	Successful sending was not dependent on characteristics recruiter, thus no influence of mixing behaviour on number of recruitees and wave reached in recruitment trees.									
	S6	1	4	0.1, 0.2,…, 3.9	0.1–3.4*	0.40										
	S7	1	4	0.1, 0.2,…, 3.9	0.1–3.4*	0.60										
	S8	1	4	0.1, 0.2,…, 3.9	0.1–3.4*	0.80										
	S9	1	4	0.1, 0.2,…, 3.9	0.1–3.4*	1										
Assuming a beta-binomial distribution with lowest possible ***σ***^***2***^	S10	1	4	0.1, 0.2,…, 3.9	0.1–1.1^Є^	0.19										
	S11	1	4	0.1, 0.2,…, 3.9	0.1–1.1^Є^	0.40										
	S12	1	4	0.1, 0.2,…, 3.9	0.1–1.1^Є^	0.60										
	S13	1	4	0.1, 0.2,…, 3.9	0.1–1.1^Є^	0.80										
	S14	1	4	0.1, 0.2,…, 3.9	0.1–1.1^Є^	1										
Differences in mixing behaviour	S15	100 random seeds from data	4	See S3 Table (+0.6)	see S3 Table (-0.6)	1	Assumed heterogeneity in mixing behaviour for different waves, as observed in the data. Values taken from Table 2.									
	S16	100 random seeds from data	**4**	see S3 Table (+0.6)	see S3 Table (-0.6)	1	0.50	0.50	0.33	0.33	0.33	0.33	0.33	0.33	0.50	0.50
	S17	100 random seeds from data	**4**	see S3 Table (+0.6)	see S3 Table (-0.6)	1	1	0.50	0.33	0.33	0.33	0.33	0.33	0.33	0.50	1
Active seeds	S18	100 highly active seeds	**4**	see S3 Table (+0.6)	see S3 Table (-0.6)	1	1	0.50	0.33	0.33	0.33	0.33	0.33	0.33	0.50	1

*High *σ*^*2*^ chosen, similar to observed in data. See S2A Fig for the different shapes of the beta-binomial distribution.

^Є^Lowest possible *σ*^*2*^ with respect to chosen *μ*. See S2B Fig for the different shapes of the beta-binomial distribution.

In scenarios S2 to S4, we ran 1000 simulations with 1015 seeds each, with characteristics (sex, age group and education level) and recruitment patterns as observed in the data. One set of seeds together with their recruitment trees was interpreted as one simulated data set, which could be compared to the observed data set. In scenario S2, the mean number of sent invitations and probability of acceptance were stratified by recruiter characteristics, with overall mixing proportions as observed in the data. In scenario S3, probabilities of sending and acceptation were stratified by recruiter characteristics, and differed between seeds (wave 0) and all recruitees in waves 1 to 6. In addition, in scenario S4, we used mixing proportions stratified by waves (Table 2).

Next, we defined 10 scenarios (S5 to S14) to study conditions for obtaining large recruitment trees, and the influence of increased recruitment on the total number of recruitees and maximum wave number reached by recruitment. Per scenario, we varied the mean number of invitations sent out by individuals (μ) between 0.1 and 3.9, and we varied the probability of acceptance of an invitation between 0.19 (as observed in the data), 0.40, 0.60, 0.80 and 1. In total this led to 10 scenarios with different recruitment probabilities, where recruitment probability was not stratified by characteristics of the recruiter. In scenarios S5 to S9, we defined *σ*^*2*^ based on the sample variance of the number of sent invitations observed in the data; for higher values of *μ*, the observed sample variance was not compatible with the beta-binomial distribution; we then used the maximum value of *σ*^*2*^ possible. We used a high value of *σ*^*2*^ to maintain in each set of simulations a bimodal distribution of the number of invitations sent out, as observed in the data. To investigate the influence of other than bimodal distributions of number of invitations sent out, in scenarios S10 to S14 the *lowest* possible value of *σ*^*2*^ compatible with a given value of *μ* was used. The shapes of the beta-binomial distributions used in scenarios S5-S14 are shown in S2 Fig.

To investigate how differences between recruiters and assortative recruitment can be exploited to increase the number of recruitees and waves, and how this affects the sample composition, we performed simulations for an additional four scenarios (S15 to S18). For each scenario, we assumed that each type of recruiter sent out on average $\bar{x}$ + 0.6 invitations. This value was chosen to ensure that the mean number of successfully sent invitations was above 1 for all types of recruiters (see S3 Table). The variance of number of invitations was set to s2–0.6, to ensure compatibility with the beta-binomial distribution. The probability of acceptance was set to 1. In scenario S15, we used mixing proportions stratified by waves as observed in the data. We then compared random mixing by characteristics (S16) with a situation where females recruit only females and academics only academics (S17). In scenarios S15 to S17, we used 100 seeds, whose characteristics were matched to 100 randomly drawn seeds from the observed data set. Again, we interpreted one set of 100 seeds together with simulated recruitment trees as one simulated data set. In the last scenario (S18), we used the mixing proportions described in scenario S17, but started with 100 seeds, whose characteristics matched seeds from the data set who had the highest observed mean numbers of sent invitations.

## Results

### Probability of a large recruitment tree

**Fig 1:**
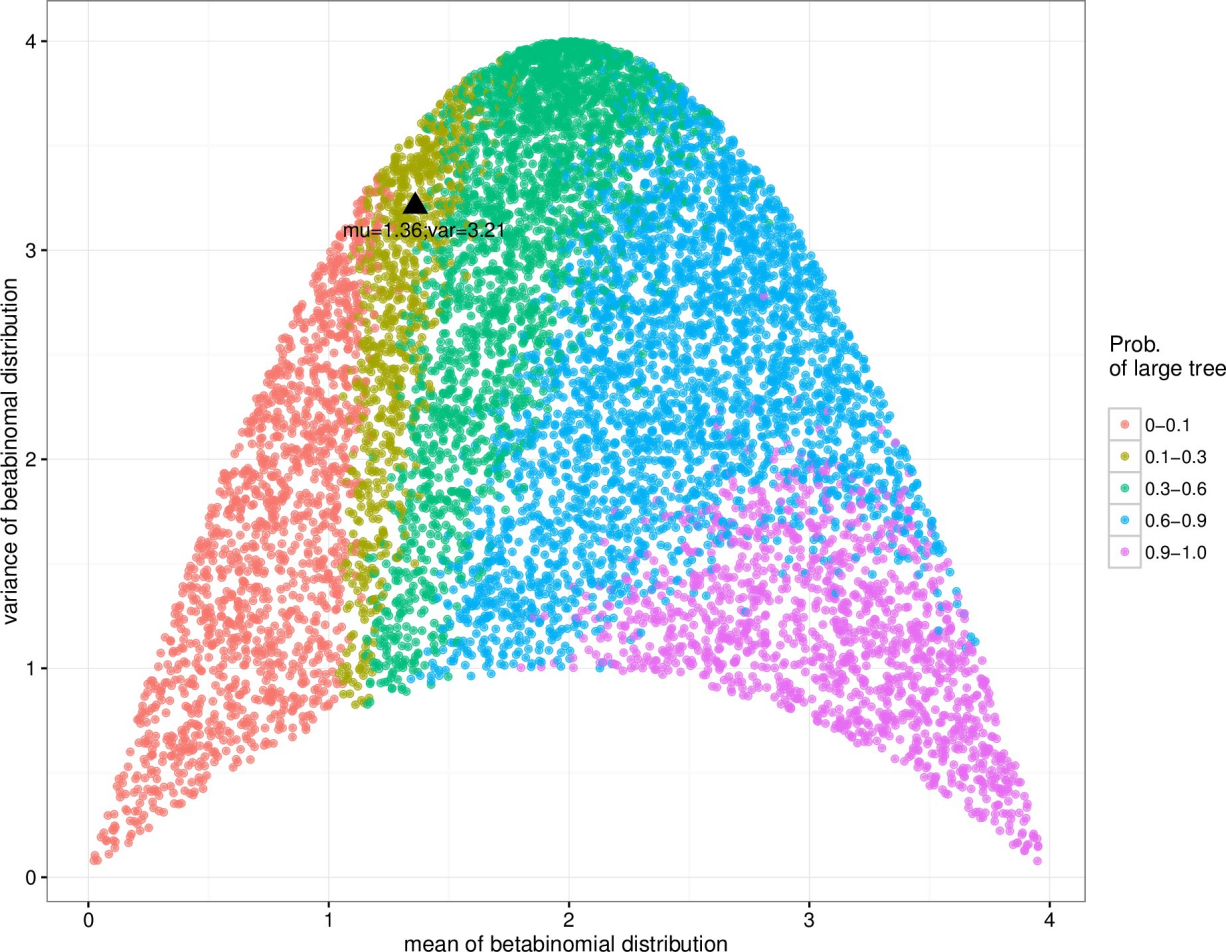
Relation between numbers of invitations and proportion of large recruitment trees. For 9000 randomly chosen combinations or *μ* and *σ*^*2*^ for the beta-binomial distribution for number of invitations we ran 100 simulations (scenario S1). We then calculated the proportion of simulations runs that resulted in a large recruitment tree (N ≥ 1000 recruitees) under the assumption of an acceptance probability of 1. The black triangle indicates the $\bar{x}$ and *s*^*2*^ of numbers of invitations sent out by participants in the data set collected in the Netherlands.

Fig 1 shows the proportion of large trees for scenario S1. The domain in the parameter region, for which results are plotted, is determined by the range of possible combinations of *μ* and *σ*^*2*^ of the beta-binomial distribution. In general, the probability of getting a large recruitment tree increases with increasing *μ*. There is a parameter region where an increase of *μ* leads to a decrease in the probability of large trees. This is the case for *μ* around 3 and *σ*^*2*^ equal to 2. This may suggest that, for some parameter combinations it is not necessary to achieve the highest possible number of invitations for maximal success of peer recruitment for a fixed variance. In this parameter region, increasing *μ* may result in a lower probability of getting a large recruitment tree. However, for a fixed variance, increasing the mean is only possible if more weight of the distribution is moved to the extremes, i.e., the probability of sending either zero or *c* invitations increases while probabilities of the values in between decreases. The extinction probability in such a situation is larger than when probability weights are distributed more evenly over the possible values of number of sent out invitations (see S2 Fig for different shapes of the beta-binomial distribution). Note that a beta-binomial does not include distributions with very small values of *σ*^*2*^ for all values of *μ*.

### Simulation runs based on empirical data

**Fig 2:**
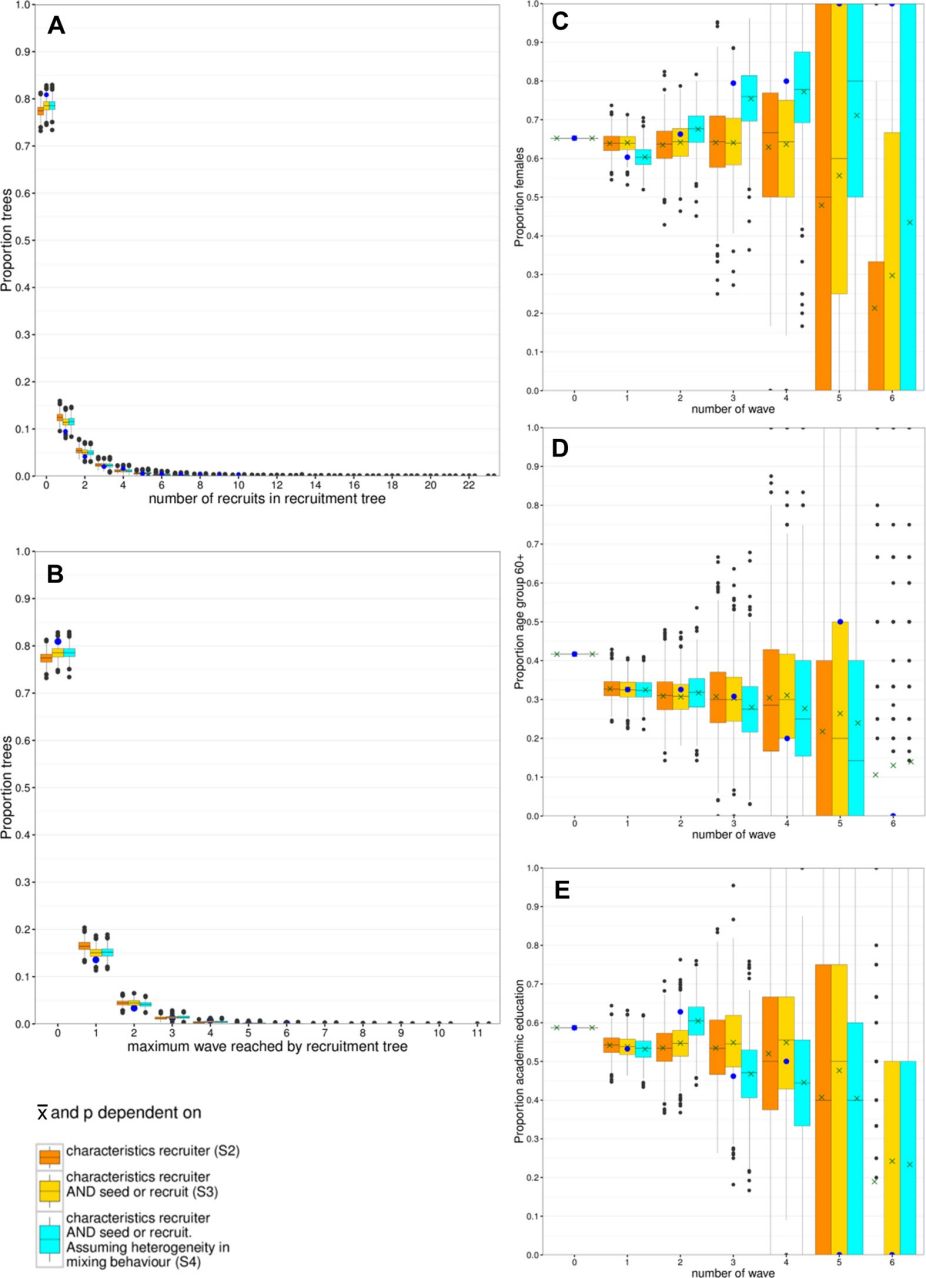
Simulation runs based on empirical data. Starting with a set of 1015 seeds with characteristics as in the empirical data, we performed 1000 simulation runs. One set of seeds together with their recruitment trees was interpreted as one simulated data set, which could be compared to the observed data set. The boxplots show the variability among simulated data sets, each boxplot consists of 1000 simulated points. The legend refers to scenarios S2 to S4 described in Table 3. The blue dots indicate the observed data. (**A**) Number of recruitees in recruitment trees in one simulated data set; (**B**) wave reached by recruitment trees in one simulated data set. Plots C to E show, for each wave, the variability in composition of each simulated data set, with (**C**) proportion of females, (**D**) age group 60+ and (**E**) having an academic education. A green cross indicates, for each wave, the mean proportion over all simulated data sets.

In scenarios S2 to S4 we compared the simulations with the observed data, to explore how well the model with the chosen parameter values could reproduce observed data. Our model slightly underestimated the number of recruitment trees with zero recruitees, and overestimated the number of trees with one and two recruitees, and one and two waves (see Fig 2A and 2B). By taking into account that seeds sent out a lower mean number of invitations than recruitees in consecutive waves (scenario S4), the model estimations slightly improved as compared to only taking recruiters’ characteristics into account (scenario S3). This suggests that seeds recruited recruitees who were more motivated than themselves to participate and to invite others. The remaining discrepancy between the simulations and the observed data, is most likely due to a practical issue during the data collection. The online software system used for sending invitations was only able to register the number of invitation letters that participants requested for further use via the survey web site. However, no information is available on whether participants actually used those requested invitation letters. If participants did not use all the invitation letters they requested, the mean number of invitations actually sent out during the data collection is lower than the mean number of requested invitation letters. The actual mean number of invitations sent out is therefore lower than estimated from the data. Using a lower value of $\bar{x}$ in the simulations would reduce the number of seeds in the model who successfully recruit recruitees and would thus improve the agreement of model results with data.

Simulated proportions of characteristics agreed with observed data in the first four waves with respect to all three variables (see Fig 2C–2E). For the remaining waves, the numbers were too small to obtain good estimates. For the first few waves, and for females in waves 0 to 4, the simulated mean proportions were in better agreement with the observed data when using mixing proportions stratified by waves (scenario S4), instead of overall mixing proportions (scenarios S2 and S3). This illustrates the influence of heterogeneity in mixing behaviour over different waves on the sample composition.

### Increasing successfully sent invitations

**Fig 3:**
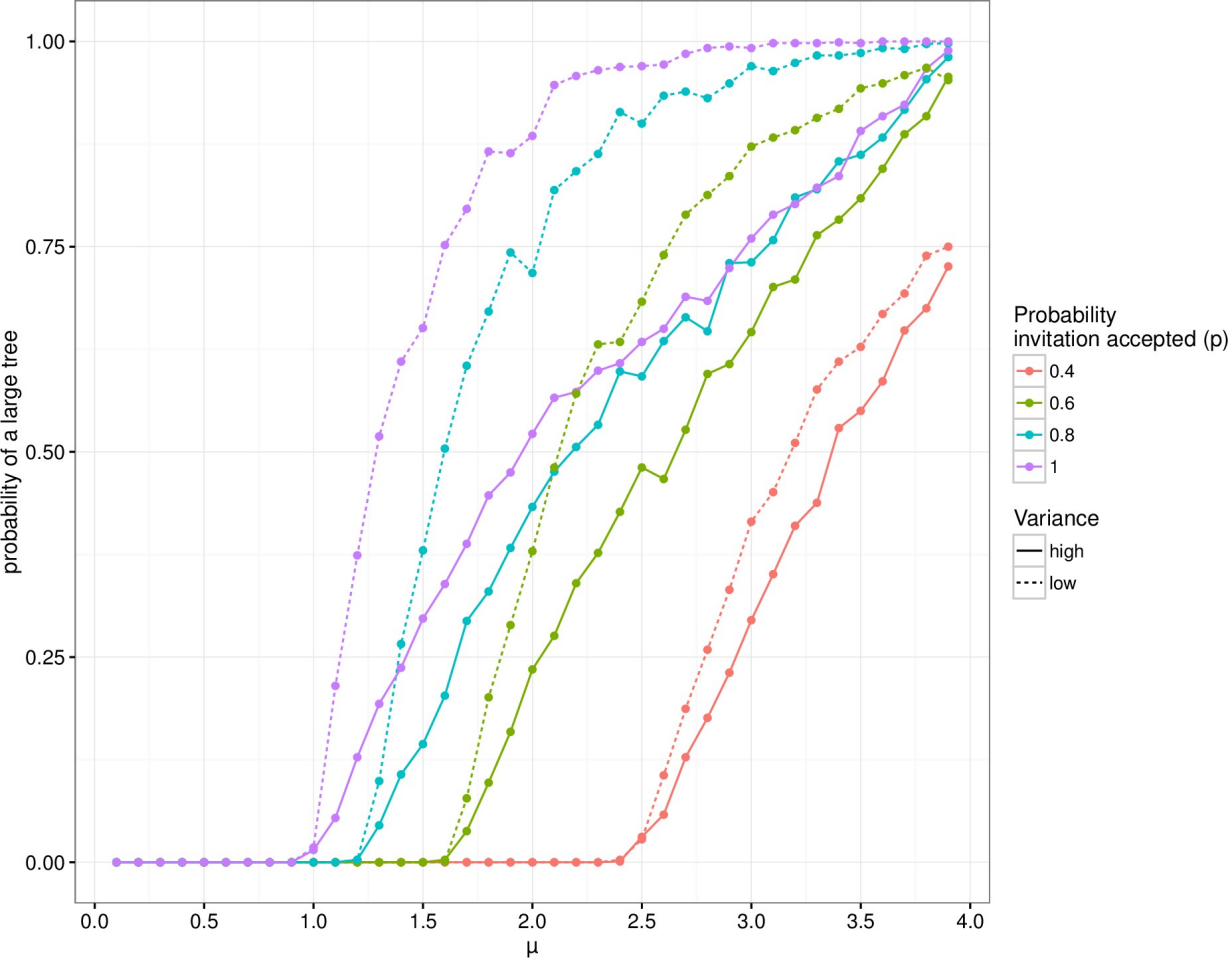
Increased number of invitations sent and probability of acceptance. Solid lines show probability of a large tree for different values of *μ* and a high *σ*^*2*^, as observed in the data (scenarios S5 to S9). The dashed lines show the same, but now assuming the lowest possible *σ*^*2*^ for each *μ* (scenarios S10 to S14).

In general, an increased mean number of invitations in combination with an increased probability of acceptance led to a higher probability of obtaining large recruitment trees (scenarios S5 to S9). The proportion of 0.19 that accepted an invitation as observed in the data set (used in scenario S5), was too low to reach a value of successfully sent invitations above 1, even when all recruiters sent out the maximum number of 4 invitations. For probabilities of acceptance between 0.40 and 1, the probability of a large tree was below 5% when the mean number of successfully sent invitations was approximately 1, but above 5% for larger mean values of acceptance probability. When assuming a bimodal distribution for the numbers of invitations sent out (as assumed in scenarios S5 to S9), with peaks at zero and four, the probability of acceptance seems to be of less importance for obtaining large recruitment trees, especially for values for *μ* of 2.4 and higher with acceptance probabilities of 0.8 and 1, as the (solid) lines overlap for larger p (Fig 3).

We further investigated a situation where 60% of all invitations sent out are accepted (scenario S7), to explore the influence of increased successful recruitment on the distribution of the number of waves reached in recruitment trees. Around a mean number of successfully sent invitations of 1, the number of waves in a tree at the end of the simulation ranged from 0 to 35. Increasing the number of successfully sent invitations resulted in lower numbers of waves reached by recruitment trees (S3 Fig). This can be explained by the termination of recruitment when a number N of recruitees was exceeded. If more recruitees enter the sample in each wave, the recruitment tree becomes wider and reaches N with a smaller number of waves.

Remarkably, when we assumed that more participants send out 2 or 3 invitations than 0 or 4, i.e., if the beta-binomial distribution was unimodal (scenarios S10 to S14), the mean number of invitations can be lower to reach the same probability of a large recruitment tree (Fig 3, and S2B Fig in supplementary information). This holds for simulations where the probability of acceptance of an invitation was at least 0.4. Fig 3 also shows, in situations with a unimodal beta-binomial distribution, that the probability of obtaining a large tree can approach 1 for situations with a high mean μ and a probability of invitation acceptance of at least 0.8. Furthermore, the probability of acceptance seems to influence the probability of obtaining a large tree for all values of *μ* (i.e., the dashed lines do not overlap in Fig 3). Our simulations suggest that for a given mean number of invitations it is more effective to motivate participants to send out at least one invitation, compared to a situation where most participants do nothing and a smaller group sends out *c* invitations.

### Exploiting asymmetric differences in recruitment

**Fig 4:**
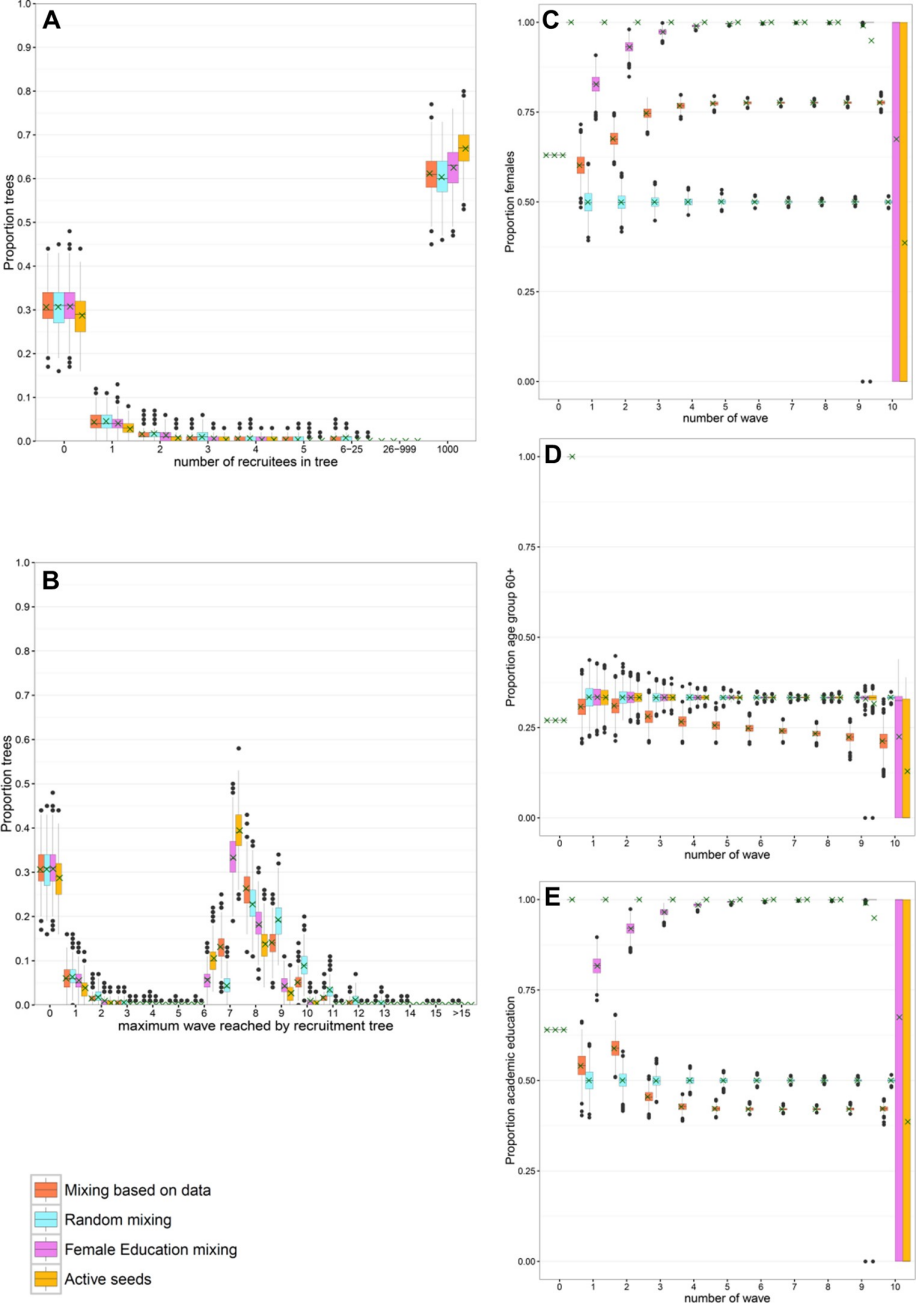
Scenario analyses of mixing behaviour. We ran simulations starting with 100 seeds with characteristics randomly drawn from the observed data (scenarios S15 to S17) and 100 active seeds (scenario S18), 1000 runs per seed. The boxplots show the variability between runs. The legend refers to scenarios S15 to S18 described in Table 3. (**A**) Number of recruitees in recruitment trees in one simulated data set; (**B**) wave reached by recruitment trees in one simulated data set. Plots C to E show, for each wave of large recruitment trees, the variability in composition of each simulated data set, with (**C**) proportion of females, (**D**) age group 60+ and (**E**) having an academic education. A green cross indicates, for each wave, the mean proportion over all simulated data sets.

In scenarios S15 to S18, we explored how differences in who mixes with whom can be used to reach a higher proportion of large recruitment trees, and how this affects the sample composition. Increasing the probability that females only recruit females and academics only recruit academics (scenario S17) slightly increased the number of recruitees, and reached large recruitment trees within a lower number of waves, compared to simulations based on the data (scenario S15) and random mixing parameters (scenario S16). Large recruitment trees were reached even faster when simulations were additionally started with 100 active seeds (scenario S18; see Fig 4A and 4B).

With random mixing (scenario S16: no correlation between recruiter and recruitee), an equilibrium was reached with a lower number of waves than when mixing proportions were stratified by waves as observed in the data (scenario S15), or mixing where participants had an increased preference for recruiting recruitees with the same characteristics (scenario S17; see Fig 4C–4E). With random mixing (scenario S16), the mean proportions of the three characteristics, over all simulated data sets, did not change more than 2% after wave 1. For mixing behaviour based on the data (scenario S15), and mixing behaviour where participants have a strong preference to invite similar others (scenario S17), equilibrium was attained at wave 4 for the proportions female and academic education. However, in scenario S15, no equilibrium was reached within 10 waves for the age group of 60 years and older. If simulations started with a random sample of seeds (scenario S16), strong assortative mixing of females and participants with an academic education eventually led to proportions of 1 in the sample (i.e., recruiters only invited recruitees with the same characteristics). If simulations started with seeds with these characteristics (scenario S18, with mixing parameters of 1 for females and academic education), the sample only included participants with the same characteristics.

**Fig 5:**
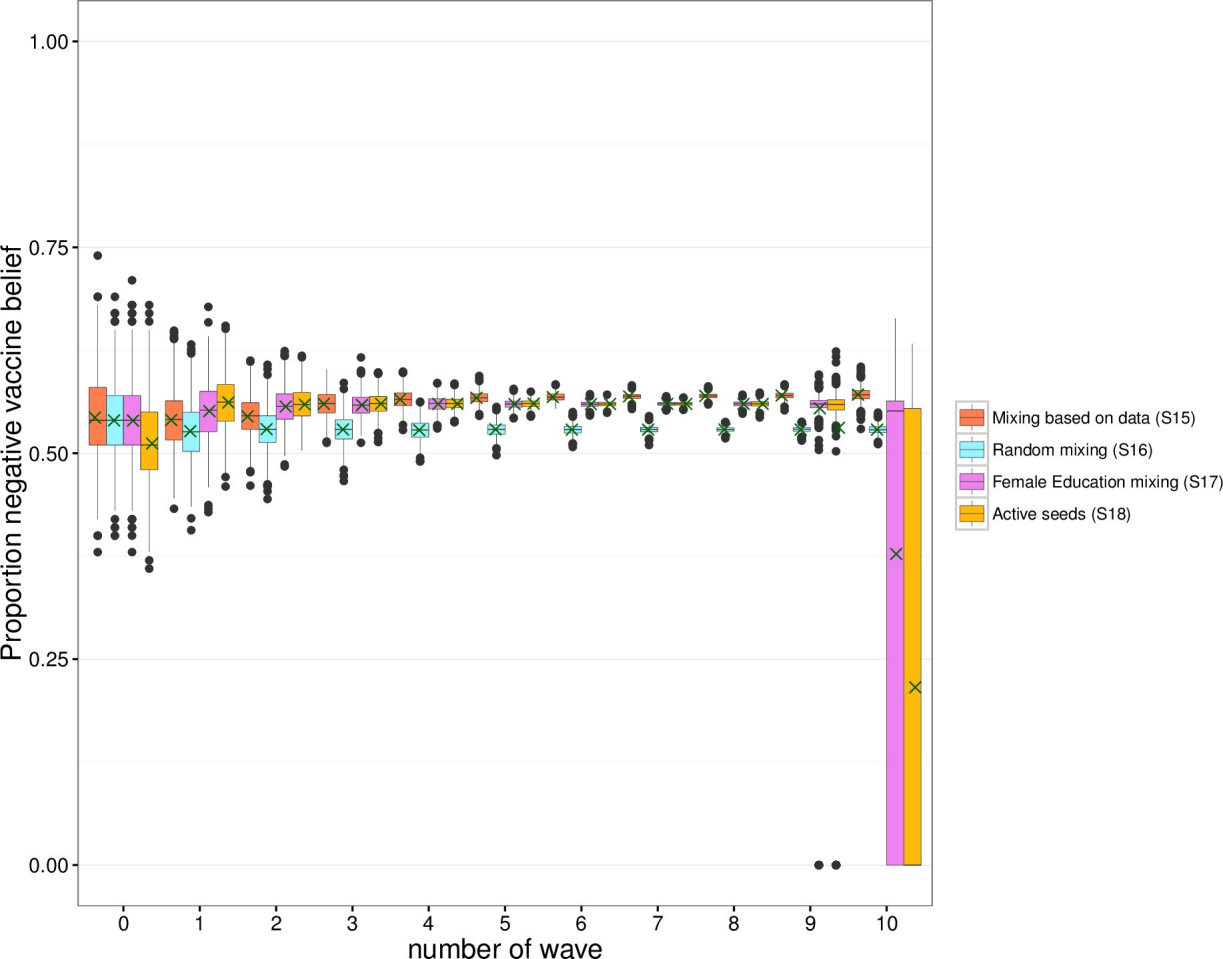
Composition per wave for negative vaccine beliefs. We ran simulations starting with 100 seeds with characteristics randomly drawn from the observed data (scenarios S15 to S17) and 100 active seeds (scenario S18), 1000 runs per seed. The boxplots show the variability between runs. The legend refers to scenarios S15 to S18 described in Table 3. A green cross indicates, for each wave, the mean proportion over all simulated data sets.

The mean proportion of negative vaccine beliefs in each wave did not change more than 2% after wave 1 for simulations based on mixing behaviour as observed in the data (scenario S15; see Fig 5). With random mixing (scenario S16), preferred mixing (scenario S17), and with simulations additionally started with active seeds (scenario S18), equilibrium was attained at wave 1. The logistic regression analysis showed significant influence of sex and age on having a positive or negative vaccine belief (see S6 Table). However, the changes over waves in the combined characteristics of individuals (e.g., the number of females in the age group 60 years and older with an academic education) were small, and therefore did not lead to large differences over waves in the proportion of individuals with a negative vaccine belief.

## Discussion

This is the first study, to our knowledge, where a simulation model and empirical data are used to analyse factors specifically important for the success of online respondent-driven recruitment. We formulated the recruitment process as a simulation model and used empirical data to quantify parameters. We included heterogeneous recruitment behaviour into our model that depended on individual characteristics. By analysing the impact of changes in model parameters on recruitment, we were able to investigate thresholds for successful peer recruitment and obtain evidence based guidance for future implementation of online RDD.

One main finding is that for some parameter combinations, it is more effective if participants send out any number of invitations between 1 and 4 for reaching large recruitment trees, than a situation where the majority of participants does nothing and a low proportion sends out all four invitations. Also, in the former situation, the probability of acceptance appeared to be of relevance for obtaining large recruitment trees for all values of the mean number of invitations. In the latter situation, the probability of acceptance seemed to be of less importance, especially for high values of the mean. In the observed data, the probability of invitation acceptance by invitees (0.19) was too low, and even with increasing the mean number of invitations to a maximum value of 4, large recruitment trees could not be obtained. Here, the average number of successful invitations stayed below 1, and therefore only small recruitment trees occurred.

We explored the influence of different mixing behaviour on the recruitment process, by choosing a combination of parameter values that led to mean numbers of successful invitations above the threshold. With assortative mixing, and by starting with seeds active with sending invitations, large recruitment trees are reached faster, within a lower number of waves, compared to random mixing. Nevertheless, assortative mixing (e.g., females only invite other females) led to samples with an overrepresentation of participants with specific characteristics, and equilibrium is reached slower, compared to random mixing. In practice, random recruitment (i.e., participants invite persons randomly from their total pool of contact persons) is difficult to ensure [16, 23], but not necessary in case RDD is used for finding other cases [2]. The observed data showed correlations for the three characteristics that increased over consecutive waves. This indicates a tendency of recruitees to ‘copy’ the recruitment behaviour of their recruiters. When using RDS estimators, which are often based on a Markov chain model, to estimate proportions of population characteristics, recruitees with certain characteristics who have a higher probability of being recruited receive a larger weighting factor. However, a violation of the first-order Markov process not only influences the point at which equilibrium is reached (as our simulations showed), it can also influence RDS estimations of the population composition [15].

Parallels exist between modelling the peer recruitment process and the spread of infectious diseases. An analogy is that the recruitment process takes places in waves, which are comparable with generations of infected in an epidemic process. We simulated the effect of an increased number of invitations sent and probability of acceptance on the probability of obtaining a large recruitment tree. This is in essence the probability of a major outbreak in a multi-type branching process, where the average number of accepted invitations determines the reproduction number of the branching process. We showed that large recruitment trees were reached more often when starting with active seeds. This is similar to stochastic epidemic models where a large outbreak of an infectious disease is more likely to occur if the index case is a high-risk individual or a superspreader [24]. A difference with transmission models is that in our peer recruitment model individuals have a maximum number of invitations and are therefore restricted in the number of individuals that they can invite, while in transmission models the number of secondary infections is not limited.

Our simulations were based on a data set collected during an online RDD survey. During this study, participants were asked to invite contact persons they had physically met in the past two weeks. Assuming that participants invited contact persons whom they actually met, the simulation results are relevant for online RDD studies that sample contact networks relevant for the transmission of respiratory pathogens. A comparison with a typical RDS study would not be justified, since the ratio of seeds to recruitees is different in RDS data sets with the aim to obtain accurate population estimates. A future RDD survey requires a combined approach to reach ongoing peer recruitment and large recruitment trees. When the mean number of invitations sent out by participants is limited, it is important that the acceptation of invitations is high. More information is needed on how to improve peer recruitment in practice. One way to learn more about ways to stimulate peer recruitment is to ask participants directly about the reasons why they do or do not invite others, e.g., as part of a questionnaire or in a follow-up study. However, this will result in information from those participants who are inclined to participate and invite others, and is therefore biased. It would be particularly important to obtain information about individuals who do not participate, but this is obviously much more difficult as they do not take part in the first place. A similar approach is to conduct proper formative research before the actual data collection, to choose the best way to invite others and the most appealing (monetary) incentives, but also by sending reminders and using technical innovations to make participation as easy as possible [25, 26].

It should be kept in mind that models always represent a simplification of reality. We assumed that the probabilities of accepting an invitation by invitees were dependent on the characteristics of the recruiter, not on those of the invitee. This adds uncertainty to our simulations, and may be one the factors why the simulation results are not fully in agreement with the observed data. We require more information on the invitees who did not participate. Such information can be collected by asking the participants more details about the persons they invite. The other limitation concerns the data itself, these were just one realization of the recruitment process and therefore provide limited information about the process in reality. Furthermore, we used data from an *online* survey, which limits the generalizability of our findings to *offline* respondent-driven studies. Moreover, during this online RDD survey, seeds were recruited from participatory surveillance panels that were not representative for the general Dutch population in terms of sex, age and educational level. We did perform two other pilot studies using RDD [27, 28], but both samples consisted of small numbers of participants and involved mainly university students of one age group.

In our model, the maximum number of invitations was kept constant (*c* = 4) to reflect the present practice in online RDD as closely as possible. A larger number of maximum invitations per recruiter will generally lead to a larger number of invitations sent out [13], but the extent to which this happens is unknown. The marginal benefits of setting a higher number of invitations are likely to be decreasing, as the number of invitations is also dependent on the number of family members, friends and acquaintances that a recruiter is able to invite. Some participants may have few close contact persons who they can invite, while others have many.

Although the branching process describing recruitment may also be studied using an analytic approach (e.g., Fig 1 can be reproduced using an analytic solution), we chose to use a simulation model to be able to include population heterogeneity. Although a simulation model does not provide exact analytic results, it is more flexible for incorporating heterogeneity and correlations between model variables. To illustrate model applicability, we included vaccine belief in the model to investigate the influence of RDD on the proportion of individuals with negative beliefs in each wave. Any other individual characteristic could be added to the model in a similar way, in order to understand the influence of recruitment behaviour on the sample composition. In a next step, we plan to add more complexity to the model by considering, among others, more covariates of participants (e.g., number of contact persons, infection status, and the behaviour of participants towards prevention programs).

By combining a simulation model with empirical data, we were able to explore the conditions for obtaining large recruitment trees and to investigate how the size and structure of recruitment trees are influenced by heterogeneous recruitment behaviour of participants. The presented model is a helpful tool that can assist public health professionals with preparing research or contact tracing using online RDD. In particular, the simulation model can provide input on the required mean number of successfully sent invitations to reach large recruitment trees, a certain sample composition or a certain number of waves.

## Supporting information

S1 Text Model formulation. (PDF)

S1 Fig **Beta binomial distributions stratified by seeds (left figure) and recruitees (right figure), and by recruiters’ characteristics.** The coloured lines indicate the different types of recruiters; the means of seeds and recruitees are indicated with the dashed lines. See S2 Table for the corresponding values observed in the data.
(TIF)

S2 Fig Shapes of the beta-binomial distributions. (**A**) Beta-binomial distributions for different *μ* and with a high *σ*^*2*^, similar to observed in the data (**B**) Beta-binomial distributions for different *μ* and with the *minimum* possible *σ*^*2*^. The values in the plot show for each line the *σ*^*2*^.
(TIF)

S3 Fig Influence on the maximum wave reached by recruitment trees. (TIF)

S1 Table Mean number of successfully sent invitations, stratified by recruiter’s characteristics, as observed in the data set. (PDF)

S2 Table Successful sending observed in the data set, stratified by seeds and recruitees, and by recruiter’s characteristics. (PDF)

S3 Table Parameter values for scenarios S15 to S18. (PDF)

S4 Table Individuals with positive or negative beliefs as observed in Dutch sample. (PDF)

S5 Table Output logistic regression for vaccine beliefs in Dutch sample. (PDF)

S6 Table Estimated probability of an individual having a positive or negative vaccine belief. (PDF)
