# Supplementary material for: A stochastic simulation model to study respondent-driven recruitment
Source: PLoS One. 2018 Nov 15;13(11):e0207507. doi: 10.1371/journal.pone.0207507 (PMC6237413; doi:10.1371/journal.pone.0207507)
Supplement: S1 Text — (PDF) [file pone.0207507.s001.pdf]

S1 Text. Model formulation

We considered a respondent-driven recruitment process, and modelled it as a *multi-type discrete time branching process*. The process can be described as follows. Let W denote the wave, i.e., the number of steps the branching process has completed up until that moment. At each wave, there are recruiters that will invite new individuals. By definition, we let the process start from $W = 0$. In this initial wave, we sample individuals from the population, and use them as *seeds* to initiate the recruitment process (i.e., seeds are the initial recruiters). Each seed can invite a number of individuals to join the study, after which then each invitee may decide to accept the invitation or not. Those that accept, the so-called 'recruitees', form wave W=1 and may then proceed to invite new individuals. Those new recruitees will end up in wave $W = 2$. This recruitment process is repeated until wave $W = w_{max}$. In our model, w_{max} was determined either by extinction of the process or by if the total number of recruitees exceeded a given bound *N*; set to 1000 recruitees.

The branching process depended on the following factors:

- *The characteristics* $\mathbf{R}$ *of the recruiters*. The recruiters and their recruitees come from a heterogeneous population, i.e., a population containing individuals with different characteristics. In general, a participant i is characterized by a vector $\mathbf{R}_i = (R_{i1}, \dots, R_{iq})$, where $R_{i1}, \dots, R_{iq}$ are q covariates for participant i. In our simulation model, we consider covariates in the form of sex, age groups and education level (as categorical variables). Sex included two categories (females, males), age groups three categories (0-39 years, 40-59 years, and 60 years and older) and educational level two categories (lower than academic education and academic education).
- *The maximum number of invitations per recruiter* c $(c = 0,1, \dots)$. c is a constant that is equal for all recruiters and for all waves.
- *The number of invitations sent out per recruiter* J $(j = 0,1, \dots, c)$. The number of invitations is a random variable that takes values between 0 and the maximum c. The number of invitations depends on sex, age and education of the recruiter.
- *The number of accepted invitations per recruiter* M $(m = 0,1, \dots, j)$. The number of accepted invitations is a random variable that takes values between 0 and the number of invitations j. The number of invitations accepted depends on sex, age and education of the recruiter.
- *The characteristics* $\mathbf{R}$ *of the recruitees*. The characteristics of recruitees in wave *W* are dependent on the characteristics of the recruiter in *W* - 1 to reflect correlations between recruiter – recruitee pairs in their characteristics, but assuming independence of the three characteristics.

In this study, we assume that the number of invitations sent by recruiter i, the accepted number of invitations for recruiter i and the characteristics of the recruitees are determined by the recruiter characteristics $\mathbf{R}_i$ (see "*The characteristics of the recruitees"* below). In practice, the number of accepted invitations likely also depends on the characteristics of those invited. However, no data on *all invitees* were currently available in the data set [1, 2], so we made the simplifying assumption that the accepted number of invitations depends on the recruiter characteristics only.

The number of invitations successfully sent out

We assumed that the number of invitations J_i for recruiter i follows a beta-binomial distribution with parameters α and β that depend on $\mathbf{R}_i$ of recruiter i:

$$J_i \sim Betabin(\alpha_{\mathbf{R}_i}, \beta_{\mathbf{R}_i}). \tag{1}$$

The rationale for a beta-binomial distribution is that it is capable of reproducing bimodal distributions that were observed in the data: the most frequently observed number of invitations occurred at zero and at the maximum value c, with any number of invitations in between occurring much less often. The corresponding probability density function is given by:

$$P(J_i = j | c, \alpha_{\mathbf{R}_i}, \beta_{\mathbf{R}_i}) = \binom{c}{j} \frac{B(j + \alpha_{\mathbf{R}_i}, c - j + \beta_{\mathbf{R}_i})}{B(\alpha_{\mathbf{R}_i}, \beta_{\mathbf{R}_i})}, \tag{2}$$

where B is the beta function. The expected value of J_i (which is comparable to the reproduction number of the branching process if all invitations are accepted) is:

$$E[J_i] = \frac{c\alpha_{\mathbf{R}_i}}{\alpha_{\mathbf{R}_i} + \beta_{\mathbf{R}_i}}. \tag{3}$$

The number of invitations accepted

Given that j invitations were sent, the number of invitations accepted M_i for recruiter i is assumed to follow a binomial distribution:

$$M_i \sim Bin(j, p_{\mathbf{R}_i}), \tag{4}$$

where the parameter $p_{\mathbf{R}_i}$ that describes the probability of acceptation, is dependent on the characteristics of the recruiter $\mathbf{R}_i$. The probability density function and expected value of M_i respectively are given by:

$$P(M_i = m | j, p_{\mathbf{R}_i}) = \binom{j}{m} p_{\mathbf{R}_i}^m (1 - p_{\mathbf{R}_i})^{j-m}, \tag{5}$$

$$E[M_i] = jp_{\mathbf{R}_i}. \tag{6}$$

The probability that recruiter i recruited m individuals into the sample is given by:

$$P(X_i = m | \mathbf{R}_i) = \sum_{j=m}^{c} P(J_i = j | c, \alpha_{\mathbf{R}_i}, \beta_{\mathbf{R}_i}) P(M_i = m | j, p_{\mathbf{R}_i}). \tag{7}$$

The expected number of recruitees recruited by recruiter *I* with characteristic R_i is then given by:

$$E[X_i \mid \boldsymbol{R_i}] = \sum_{m=0}^{c} m\, P(X_i = m \mid \boldsymbol{R_i}) = p_{\boldsymbol{R_i}} \frac{c\alpha_{\boldsymbol{R_i}}}{\alpha_{\boldsymbol{R_i}} + \beta_{\boldsymbol{R_i}}}. \tag{8}$$

Let N_W be the number of recruiters in wave W. The expected number of recruitees in wave $W+1$, N_{W+1}, is given by:

$$E[N_{W+1} \mid N_w] = \sum_{i=1}^{N_W} E[X_i \mid \boldsymbol{R_i}]\,. \tag{9}$$

The characteristics of the recruitees

The characteristics of $\mathbf{R}_{ik}$ of a recruitee k belonging to recruiter i are assumed to be dependent on the characteristics of i. Ideally, we would use the joint probability distribution $P(R_{ik1} = r_{ik1}, \dots, R_{ikq} = r_{ikq} \mid \mathbf{R}_i)$ to characterize this relationship. The available data were however not enough to estimate this joint distribution with sufficient precision; many covariate combinations $(R_{ik1} = r_{ik1}, \dots, R_{ikq} = r_{ikq})$ had too few, or even no, observations. To construct a credible joint probability distribution, we assume that the probability distribution of each covariate are mutually independent:

$$P(R_{ik1} = r_{ik1}, \dots, R_{ikq} = r_{ikq} \mid \boldsymbol{R_i}) = \prod_{t=1}^{q} P(R_{ikt} = r_{ikt} \mid \boldsymbol{R_i}), \tag{10}$$

where $P(R_{ikt} = r_{ikt} \mid \mathbf{R}_i)$ is the probability distribution of covariate R_{ikt}, and can be estimated straightforwardly from the data. Since we only have three covariates in our study, this equation reduces to:

$$P(Sex_{ik} = s_{ik}, Age_{ik} = a_{ik}, Education_{ik} = e_{ik} \mid \boldsymbol{R_i}) = P(Sex_{ik} = s_{ik} \mid \boldsymbol{R_i}) \times$$
$$P(Age_{ik} = a_{ik} \mid \boldsymbol{R_i}) \times P(Education_{ik} = e_{ik} \mid \boldsymbol{R_i}) \tag{11}$$

Influenza vaccine belief

As an illustration of the applicability of the simulation model, we added influenza vaccine belief as a categorical variable to the model. Hereby we assumed that vaccine beliefs are determined by individual characteristics and do not influence the recruitment process, i.e., recruiters do not invite recruitees with a specific vaccine belief. A logistic regression model was used to estimate the probability of an individual, with a certain sex, age and educational level, of having a positive or negative belief about the influenza vaccine (see supplementary S4-S6 Tables). Vaccine beliefs of individuals were determined based on random draws from the estimated probability distributions, where the probability distributions depended on the sex, age and educational level of individuals.

References

1. Stein ML, van der Heijden PG, Buskens V, van Steenbergen JE, Bengtsson L, Koppeschaar CE, et al. Tracking social contact networks with online respondent-driven detection: who recruits whom? BMC infectious diseases. 2015;15:522. doi: 10.1186/s12879-015-1250-z.
2. Stein ML, van Steenbergen JE, Buskens V, van der Heijden PG, Koppeschaar CE, Bengtsson L, et al. Enhancing Syndromic Surveillance With Online Respondent-Driven Detection. American journal of public health. 2015;105(8):e90-7. doi: 10.2105/AJPH.2015.302717.
