# Supplementary figures and images for: A stochastic simulation model to study respondent-driven recruitment
Source: PLoS One. 2018 Nov 15;13(11):e0207507. doi: 10.1371/journal.pone.0207507 (PMC6237413; doi:10.1371/journal.pone.0207507)

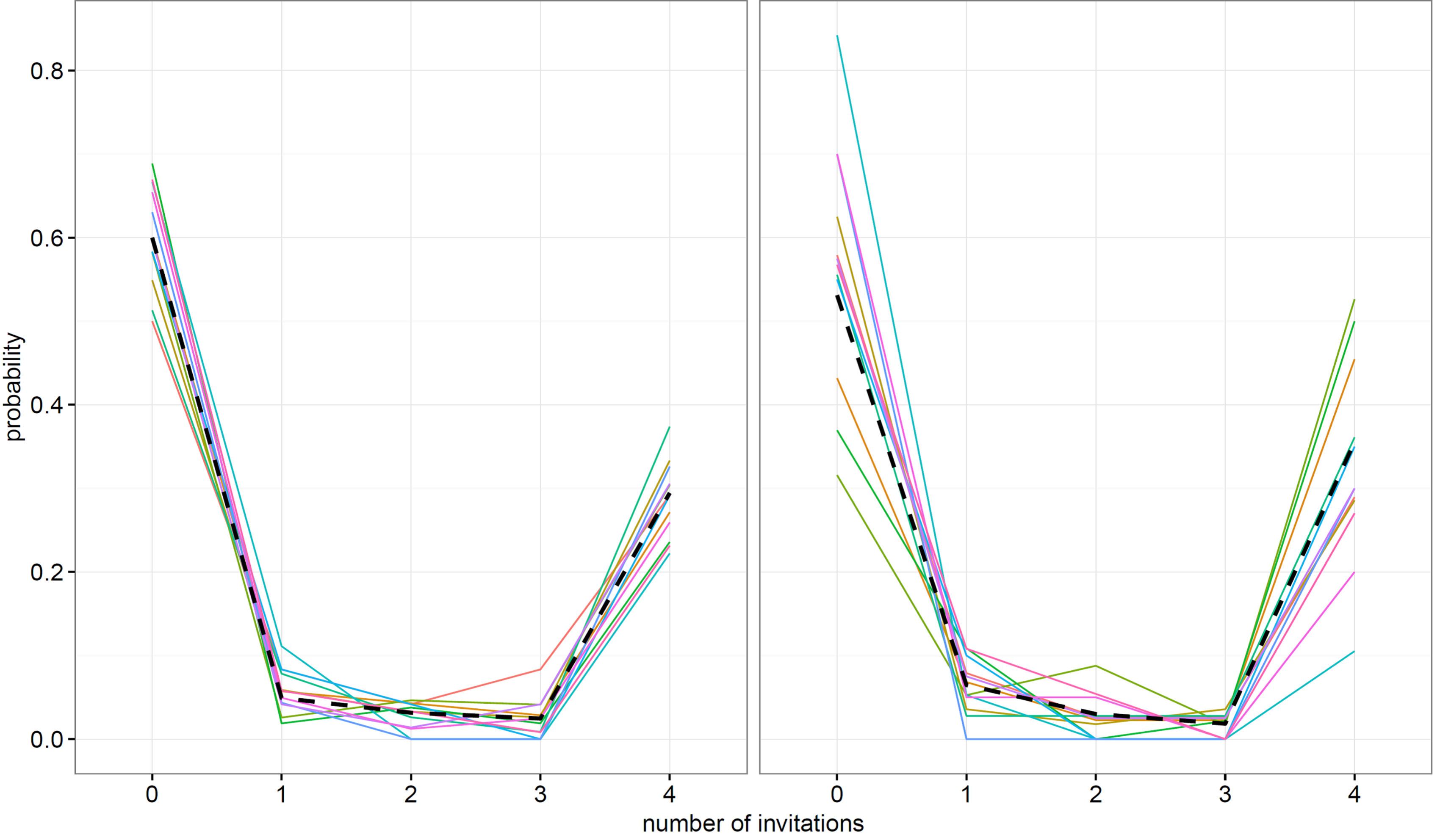

Supplement: S1 Fig — Beta binomial distributions stratified by seeds (left figure) and recruitees (right figure), and by recruiters’ characteristics. The coloured lines indicate the different types of recruiters; the means of seeds and recruitees are indicated with the dashed lines. See S2 Table for the corresponding values observed in the data. (TIF) [file pone.0207507.s002.tif]

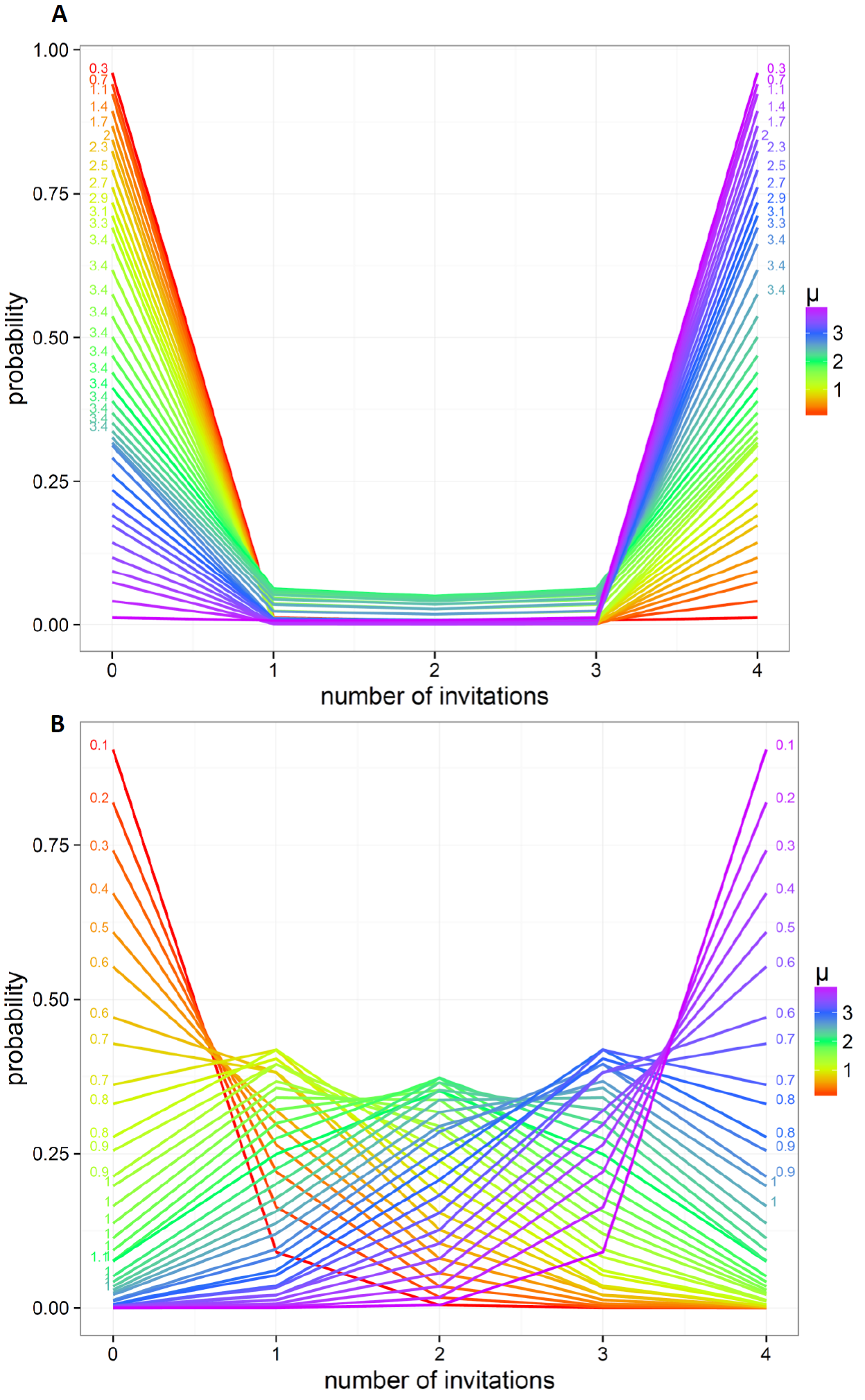

Supplement: S2 Fig — (A) Beta-binomial distributions for different μ and with a high σ2, similar to observed in the data (B) Beta-binomial distributions for different μ and with the minimum possible σ2. The values in the plot show for each line the σ2. (TIF) [file pone.0207507.s003.tif]

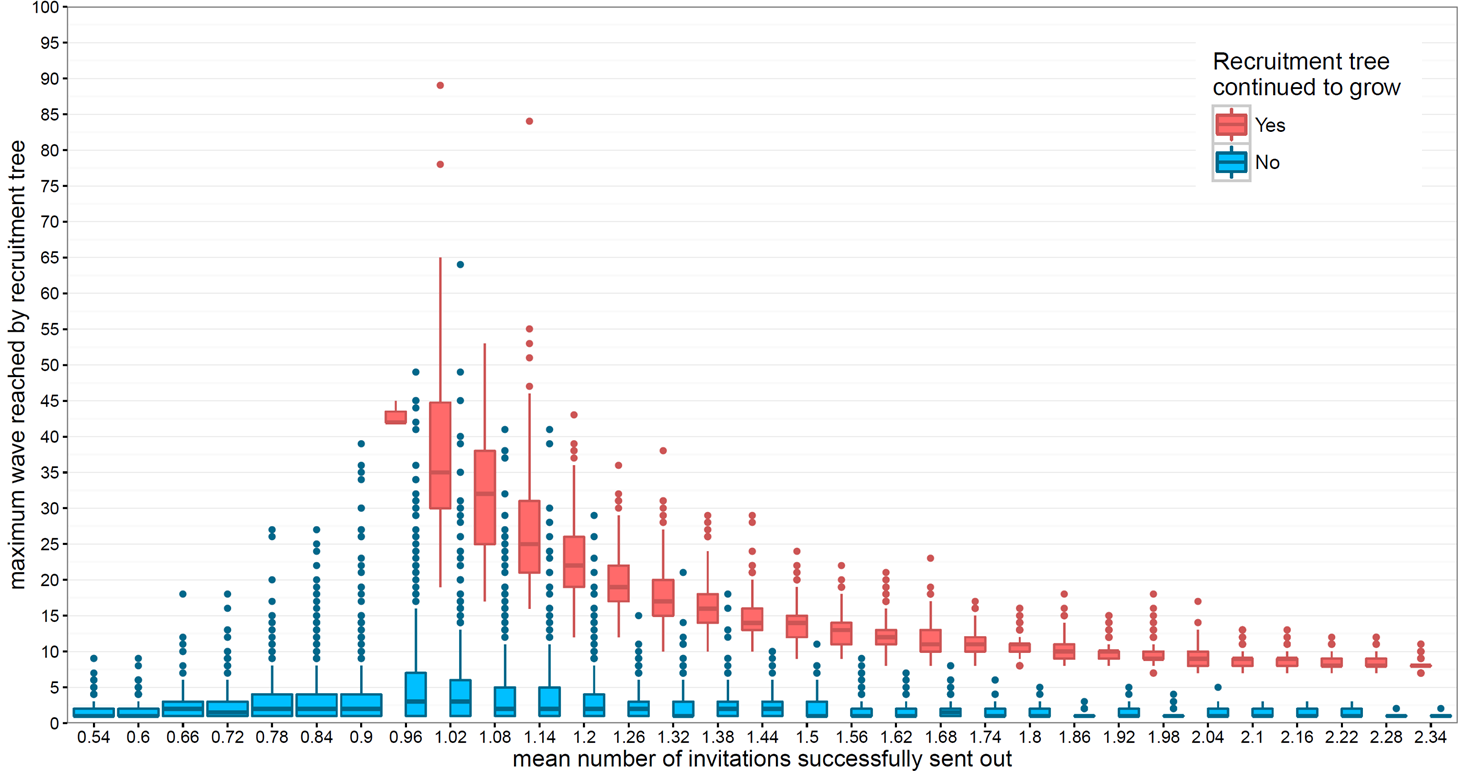

Supplement: S3 Fig — (TIF) [file pone.0207507.s004.tif]
