# Supplementary material for: A stochastic simulation model to study respondent-driven recruitment
Source: PLoS One. 2018 Nov 15;13(11):e0207507. doi: 10.1371/journal.pone.0207507 (PMC6237413; doi:10.1371/journal.pone.0207507)
Supplement: S1 Table — (PDF) [file pone.0207507.s005.pdf]

S1 Table. Mean number of successfully sent invitations, stratified by recruiter's characteristics, as observed in the data set.

Recruiter			Proportions that sent 0-4 invitations							Beta-binomial distribution			
Sex	Age group	Educational level	0	1	2	3	4	$\bar{x}$	s^2	α	β	p	$\bar{x}$ * p
F	A1	A	0.55	0.08	0.03	0.05	0.29	1.45	3.24	0.09	0.15	0.10	0.15
F	A1	B	0.54	0.06	0.04	0.03	0.34	1.58	3.45	0.07	0.10	0.20	0.32
F	A2	A	0.57	0.05	0.03	0.03	0.32	1.48	3.39	0.06	0.09	0.20	0.30
F	A2	B	0.52	0.03	0.06	0.04	0.35	1.67	3.48	0.07	0.09	0.27	0.45
F	A3	A	0.59	0.05	0.03	0.02	0.32	1.42	3.38	0.04	0.08	0.21	0.30
F	A3	B	0.52	0.07	0.03	0.01	0.37	1.64	3.55	0.06	0.08	0.16	0.26
M	A1	A	0.79	0.07	0.00	0.00	0.14	0.64	2.02	0.03	0.14	0.11	0.07
M	A1	B	0.57	0.09	0.02	0.00	0.32	1.41	3.36	0.06	0.10	0.21	0.30
M	A2	A	0.65	0.03	0.00	0.00	0.32	1.30	3.48	0.01	0.03	0.20	0.26
M	A2	B	0.59	0.05	0.02	0.04	0.30	1.41	3.34	0.05	0.09	0.16	0.22
M	A3	A	0.66	0.05	0.02	0.02	0.25	1.14	3.00	0.04	0.10	0.25	0.29
M	A3	B	0.65	0.07	0.04	0.01	0.24	1.13	2.88	0.05	0.13	0.22	0.25
							mean values	1.36	3.21			0.19	0.26
