# Supplementary material for: A stochastic simulation model to study respondent-driven recruitment
Source: PLoS One. 2018 Nov 15;13(11):e0207507. doi: 10.1371/journal.pone.0207507 (PMC6237413; doi:10.1371/journal.pone.0207507)
Supplement: S2 Table — (PDF) [file pone.0207507.s006.pdf]

S2 Table. Successful sending observed in the data set, stratified by seeds and recruitees, and by recruiter's characteristics.

	Recruiter			Proportions that sent 0-4 invitations							Beta-binomial distribution			
seed / recruitees	Sex	Age group	Educational level	0	1	2	3	4	$\bar{x}$	s^2	α	β	p	$\bar{x}$ * p
seed	F	A1	A	0.50	0.08	0.04	0.08	0.29	1.58	3.30	0.12	0.19	0.13	0.21
seed	F	A1	B	0.60	0.06	0.04	0.03	0.27	1.31	3.15	0.06	0.13	0.21	0.27
seed	F	A2	A	0.55	0.06	0.03	0.03	0.33	1.54	3.42	0.06	0.10	0.20	0.31
seed	F	A2	B	0.58	0.03	0.05	0.04	0.30	1.46	3.34	0.06	0.10	0.27	0.39
seed	F	A3	A	0.69	0.02	0.04	0.02	0.24	1.09	2.94	0.03	0.08	0.25	0.27
seed	F	A3	B	0.51	0.08	0.03	0.01	0.37	1.65	3.54	0.06	0.08	0.16	0.27
seed	M	A1	A	0.67	0.11	0.00	0.00	0.22	1.00	3.00	0.05	0.14	0.00	0.00
seed	M	A1	B	0.58	0.08	0.04	0.00	0.29	1.33	3.28	0.06	0.12	0.25	0.33
seed	M	A2	A	0.63	0.04	0.00	0.00	0.33	1.35	3.52	0.02	0.04	0.18	0.24
seed	M	A2	B	0.60	0.04	0.01	0.04	0.31	1.42	3.40	0.05	0.08	0.17	0.24
seed	M	A3	A	0.65	0.05	0.01	0.02	0.26	1.19	3.10	0.04	0.09	0.24	0.28
seed	M	A3	B	0.67	0.06	0.03	0.01	0.23	1.07	2.84	0.04	0.12	0.22	0.23
recruitees	F	A1	A	0.58	0.08	0.03	0.03	0.29	1.37	3.27	0.07	0.12	0.08	0.11
recruitees	F	A1	B	0.43	0.07	0.02	0.02	0.45	2.00	3.72	0.07	0.07	0.19	0.39
recruitees	F	A2	A	0.63	0.04	0.02	0.04	0.29	1.32	3.31	0.04	0.08	0.19	0.25
recruitees	F	A2	B	0.32	0.05	0.09	0.02	0.53	2.39	3.35	0.13	0.09	0.28	0.67
recruitees	F	A3	A	0.37	0.11	0.00	0.02	0.50	2.17	3.66	0.09	0.07	0.17	0.37
recruitees	F	A3	B	0.56	0.03	0.03	0.03	0.36	1.61	3.67	0.04	0.06	0.16	0.25
recruitees	M	A1	A	0.84	0.05	0.00	0.00	0.11	0.47	1.60	0.02	0.14	0.22	0.11
recruitees	M	A1	B	0.55	0.10	0.00	0.00	0.35	1.50	3.63	0.05	0.08	0.17	0.25
recruitees	M	A2	A	0.70	0.00	0.00	0.00	0.30	1.20	3.54	0.00	0.01	0.25	0.30
recruitees	M	A2	B	0.58	0.08	0.03	0.03	0.30	1.40	3.32	0.06	0.11	0.14	0.20
recruitees	M	A3	A	0.70	0.05	0.05	0.00	0.20	0.95	2.68	0.04	0.14	0.32	0.30
recruitees	M	A3	B	0.57	0.11	0.05	0.00	0.27	1.30	3.05	0.09	0.17	0.25	0.32
