# Supplementary material for: A stochastic simulation model to study respondent-driven recruitment
Source: PLoS One. 2018 Nov 15;13(11):e0207507. doi: 10.1371/journal.pone.0207507 (PMC6237413; doi:10.1371/journal.pone.0207507)
Supplement: S3 Table — (PDF) [file pone.0207507.s007.pdf]

S3 Table. Parameter values for scenarios S15 to S18.

	Recruiter			Proportions that sent 0-4 invitations							Beta-binomial distribution			
seed / recruitees	Sex	Age group	Educational level	0	1	2	3	4	$\bar{x}$ +0.6	s^2 -0.6	α	β	p	$\bar{x}$ * p
seed	F	A1	A	0.27	0.13	0.12	0.14	0.35	2.18	2.70	0.41	0.34	1.00	2.18
seed	F	A1	B	0.30	0.15	0.13	0.15	0.26	1.91	2.55	0.45	0.49	1.00	1.91
seed	F	A2	A	0.29	0.12	0.10	0.13	0.36	2.14	2.82	0.34	0.30	1.00	2.14
seed	F	A2	B	0.30	0.13	0.12	0.13	0.32	2.06	2.74	0.37	0.35	1.00	2.06
seed	F	A3	A	0.33	0.18	0.15	0.15	0.19	1.69	2.34	0.48	0.66	1.00	1.69
seed	F	A3	B	0.29	0.10	0.09	0.11	0.41	2.25	2.94	0.28	0.22	1.00	2.25
seed	M	A1	A	0.37	0.17	0.13	0.13	0.19	1.60	2.40	0.40	0.60	1.00	1.60
seed	M	A1	B	0.32	0.14	0.12	0.14	0.28	1.93	2.68	0.38	0.41	1.00	1.93
seed	M	A2	A	0.35	0.12	0.10	0.11	0.32	1.95	2.92	0.27	0.29	1.00	1.95
seed	M	A2	B	0.31	0.13	0.11	0.13	0.32	2.02	2.80	0.34	0.33	1.00	2.02
seed	M	A3	A	0.33	0.16	0.14	0.14	0.23	1.79	2.50	0.43	0.53	1.00	1.79
seed	M	A3	B	0.32	0.19	0.16	0.15	0.18	1.67	2.24	0.55	0.76	1.00	1.67
recruitees	F	A1	A	0.31	0.14	0.12	0.14	0.29	1.97	2.67	0.39	0.41	1.00	1.97
recruitees	F	A1	B	0.27	0.05	0.04	0.06	0.57	2.60	3.12	0.15	0.08	1.00	2.60
recruitees	F	A2	A	0.32	0.14	0.12	0.13	0.29	1.92	2.71	0.36	0.39	1.00	1.92
recruitees	F	A2	B	0.21	0.03	0.02	0.03	0.71	2.99	2.75	0.10	0.03	1.00	2.99
recruitees	F	A3	A	0.26	0.04	0.03	0.04	0.64	2.77	3.06	0.11	0.05	1.00	2.77
recruitees	F	A3	B	0.31	0.09	0.08	0.10	0.42	2.21	3.07	0.23	0.19	1.00	2.21
recruitees	M	A1	A	0.34	0.35	0.21	0.08	0.02	1.07	1.00	2.69	7.36	1.00	1.07
recruitees	M	A1	B	0.33	0.10	0.09	0.10	0.38	2.10	3.03	0.25	0.22	1.00	2.10
recruitees	M	A2	A	0.39	0.11	0.09	0.11	0.30	1.80	2.94	0.24	0.29	1.00	1.80
recruitees	M	A2	B	0.31	0.13	0.12	0.13	0.31	2.00	2.72	0.37	0.37	1.00	2.00
recruitees	M	A3	A	0.34	0.20	0.17	0.15	0.14	1.55	2.08	0.59	0.93	1.00	1.55
recruitees	M	A3	B	0.29	0.16	0.15	0.16	0.24	1.90	2.45	0.50	0.56	1.00	1.90
