# Supplementary material for: A stochastic simulation model to study respondent-driven recruitment
Source: PLoS One. 2018 Nov 15;13(11):e0207507. doi: 10.1371/journal.pone.0207507 (PMC6237413; doi:10.1371/journal.pone.0207507)
Supplement: S4 Table — (PDF) [file pone.0207507.s008.pdf]

S4 Table. Individuals with positive or negative beliefs as observed in Dutch sample.

Sex	Age group	Educational level	Positive vaccine belief	Negative vaccine belief
F	A1	A	35.5%	64.5%
		B	39.5%	60.5%
	A2	A	37.8%	62.2%
		B	48.2%	51.8%
	A3	A	43.4%	56.6%
		B	41.7%	58.3%
M	A1	A	46.4%	53.6%
		B	56.8%	43.2%
	A2	A	45.4%	54.6%
		B	42.9%	57.1%
	A3	A	59.4%	40.6%
		B	63.9%	36.1%
