# Supplementary material for: A stochastic simulation model to study respondent-driven recruitment
Source: PLoS One. 2018 Nov 15;13(11):e0207507. doi: 10.1371/journal.pone.0207507 (PMC6237413; doi:10.1371/journal.pone.0207507)
Supplement: S5 Table — (PDF) [file pone.0207507.s009.pdf]

S5 Table. Output logistic regression for vaccine beliefs in Dutch sample.

	estimate[a]	Std. Error	z value	Pr(>|z|)
Intercept	-0.555	0.150	-3.709	0.000
Rec_age_A2	0.068	0.152	0.448	0.654
Rec_age_A3	0.319	0.156	2.044	0.041
Rec_gender_M	0.434	0.113	3.835	0.000
Rec_edu_B	0.184	0.108	1.702	0.089

[a]Null deviance: 2000.2 (df: 1447); residual variance: 1971.7 (df: 1443) and AIC: 1981.7.
