# Supplementary material for: A stochastic simulation model to study respondent-driven recruitment
Source: PLoS One. 2018 Nov 15;13(11):e0207507. doi: 10.1371/journal.pone.0207507 (PMC6237413; doi:10.1371/journal.pone.0207507)
Supplement: S6 Table — (PDF) [file pone.0207507.s010.pdf]

**S6 Table. Estimated probability of an individual having a positive or negative vaccine belief.**

| Sex | Age group | Educational level | Positive vaccine belief | Negative vaccine belief |
|---|---|---|---|---|
| F | A1 | A | 0.36 | 0.64 |
| F | A1 | B | 0.41 | 0.59 |
| F | A2 | A | 0.38 | 0.62 |
| F | A2 | B | 0.42 | 0.58 |
| F | A3 | A | 0.44 | 0.56 |
| F | A3 | B | 0.49 | 0.51 |
| M | A1 | A | 0.47 | 0.53 |
| M | A1 | B | 0.52 | 0.48 |
| M | A2 | A | 0.49 | 0.51 |
| M | A2 | B | 0.53 | 0.47 |
| M | A3 | A | 0.55 | 0.45 |
| M | A3 | B | 0.59 | 0.41 |
